# Nursing under the Ministry of Health

**Published:** 1921-08-27

**Authors:** 


### THE MATRONS' AND SISTERS' DEPARTMENT.

### NURSING UNDER THE MINISTRY OF HEALTH.

Although, owing to financial pressure, the Ministry of Health can hardly yet be said to have got into its full stride, the record of the past year affords ample evidence that the value to the nation of good nursing is fully appreciated at headquarters. The figures furnished for 1920-1921 present many features of special interest to nurses engaged on public health work or under the Poor Law.

There has been much to discourage the starting of new undertakings, however greatly needed, either by voluntary agencies or municipal zeal. Yet the number of new maternity and child centres has actually risen in the year by 183, and the number of health visitors by 215. Great encouragement was given to such work by the fall of the infant death-rate in 1920 to 80 per 1,000, the lowest point yet reached, and there is every hope that the public interest in this work will go on steadily increasing. Approximately the equivalent of the whole time of 1,617 women (of whom seventy-four per cent. are trained nurses and eighty-three per cent. midwives) is devoted to health visiting. According to a standard recently adopted the services of one whole-time health visitor should be available for every 400 births, and on this average there is still a deficit in the country of some 600 health visitors. Special new courses of training for health visiting to carry out the prescribed curriculum of the Board of Education were started at King's College for Women, Bedford College for Women, Battersea Polytechnic, Liverpool School of Hygiene, and University College of South Wales, Cardiff.

The growth of midwifery is attested by the fact that midwives now attend the majority of births in England and Wales, the percentage in 1919 being fifty-one per cent. in London and sixty-nine per cent. in the county boroughs. The serious shortage of rural midwives continues, but is being bravely met by the spread of district nursing associations. Of these 184 were newly started in 1920, while but five are reported as having fallen into abeyance. Maternity accommodation has increased by 206 beds. Nurses have to be provided under maternity and child-welfare schemes for the home nursing of maternity patients, and for infectious diseases of young children. Local authorities to the number of 135 contract with a district nursing association for this service, while some of the larger councils employ a staff of specially trained nurses, particularly for ophthalmia neonatorum.

No part of the work carried out under the Ministry of Health is of more importance than that which relates to Poor-Law infirmaries. A definite stimulus has been given to the medical and nursing services of these institutions, and much effort has been expended on the task of raising the standard of efficiency throughout the country. But it would seem great care has been taken to avoid drastic or unnecessary interference with the existing system. So far as nursing is concerned, progress has been retarded by the scarcity of good candidates for training, which, though less marked in the present year, still adversely affects the standard in the less advantageously situated Unions. The age-limit has been reduced to nineteen without appreciably influencing the supply. Pressure has been put upon the Ministry to permit probationers to reside outside the nurses' home, but for reasons of "health, discipline, and *esprit de corps*" this expedient has not found favour at headquarters. The limit of possible concession, we learn, would be to consider the establishment of separate nurses' hostels.

Two more infirmaries, the Middlesbrough and West Hartlepool Unions, have been added to the list of training-schools for nurses, in each case on the appointment of a resident woman medical officer. There are now thirty Metropolitan and eighty-nine provincial Poor-Law institutions providing training which, with the possession of the C.M.B. certificate, is accepted as qualifying for the post of superintendent nurse.

Statistics from Lancashire show that in the twenty-three Lancashire infirmaries and institutions with two or more medical officers there was an average of one medical officer to every seventy-five patients, and one nurse to every six patients, while in the remaining fifteen smaller institutions in the county the averages were one medical officer to every 166 patients and one nurse to every 13.5 patients.

